# Non-insulin determinant pathways maintain glucose homeostasis upon metabolic surgery

**DOI:** 10.1038/s41421-018-0062-x

**Published:** 2018-09-25

**Authors:** Zongshi Lu, Xiao Wei, Fang Sun, Hexuan Zhang, Peng Gao, Yunfei Pu, Anlong Wang, Jing Chen, Weidong Tong, Qiang Li, Xunmei Zhou, Zhencheng Yan, Hongting Zheng, Gangyi Yang, Yu Huang, Daoyan Liu, Zhiming Zhu

**Affiliations:** 1 Department of Hypertension and Endocrinology, Center for Hypertension and Metabolic Diseases, Daping Hospital, Third Military Medical University, Chongqing Institute of Hypertension, Chongqing, 400042 China; 2 Department of Gastrointestinal Metabolic Surgery, Daping Hospital, Third Military Medical University, Chongqing, 400042 China; 3 Department of Endocrinology, Xinqiao Hospital, Third Military Medical University, Chongqing, 400037 China; 4 Department of Endocrinology, the Second Affiliated Hospital, Chongqing Medical University, Chongqing, 400010 China; 5 Institute of Vascular Medicine and School of Biomedical Sciences, Chinese University of Hong Kong, BMSB315, Shatin, Hong Kong 00852 China

## Abstract

Insulin is critical for glucose homeostasis, and insulin deficiency or resistance leads to the development of diabetes. Recent evidence suggests that diabetes can be remitted independent of insulin. However, the underlying mechanism remains largely elusive. In this study, we utilized metabolic surgery as a tool to identify the non-insulin determinant mechanism. Here, we report that the most common metabolic surgery, Roux-en-Y gastric bypass (RYGB), reduced insulin production but persistently maintained euglycemia in healthy Sprague-Dawley (SD) rats and C57 mice. This reduction in insulin production was associated with RYGB-mediated inhibition of pancreatic preproinsulin and polypyrimidine tract-binding protein 1. In addition, RYGB also weakened insulin sensitivity that was evaluated by hyperinsulinemic-euglycemic clamp test and downregulated signaling pathways in insulin-sensitive tissues. The mechanistic evidence suggests that RYGB predominately shifted the metabolic profile from glucose utilization to fatty acid oxidation, enhanced the energy expenditure and activated multiple metabolic pathways through reducing gut energy uptake. Importantly, the unique effect of RYGB was extended to rats with islet disruption and patients with type 2 diabetes. These results demonstrate that compulsory rearrangement of the gastrointestinal tract can initiate non-insulin determinant pathways to maintain glucose homeostasis. Based on the principle of RYGB action, the development of a noninvasive intervention of the gastrointestinal tract is a promising therapeutic route to combat disorders characterized by energy metabolism dysregulation.

## Introduction

Insulin has been deemed the sole hypoglycemic hormone in mammals since its discovery in 1921^1^. A generally accepted concept is that insulin is essential for maintaining glucose homeostasis and regulating carbohydrate, lipid, and protein metabolism through eliciting a diverse array of signaling pathways by binding to its specific receptor^2–4^. The pathogenesis of diabetes is characterized by the progressive development of insulin resistance and a deficiency in insulin secretion, leading to overt hyperglycemia^3–5^. Therefore, current diabetes therapies focus on insulin replenishment and insulin sensitivity improvement through medicine and lifestyle changes^5^. Outside of classical management strategies, metabolic surgery is presently thought to be the most durable intervention for the remission of diabetes and obesity^4^. Roux-en-Y gastric bypass (RYGB) is the most effective and widely performed metabolic surgery^6^. RYGB can quickly correct hyperglycemia and hyperinsulinemia, despite the presence of continued insulin resistance and obesity in patients with type 2 diabetes^7^. In addition to weight loss and glycemic control, metabolic surgery also improves the micro- and macrovascular complications of diabetes and even of cancers^8,9^. Although metabolic surgery-mediated changes in gut hormones, nutrient sensing, intestinal glucose utilization, microbiota, and bile acid metabolism in diabetes and obesity have been proposed^10–14^, the underlying mechanism remains elusive. Furthermore, several studies suggest that central leptin administration and RYGB are sufficient to restore euglycemia in the insulin-deficient state, which clearly indicates the existence of an insulin-independent hypoglycemic phenomenon, but its underlying mechanism is poorly understood^15,16^. An unsolved question is how glucose homeostasis can be maintained without the participation of insulin. One important obstacle to addressing this issue is that most studies investigate the animal models or patients with diabetes or obesity, which is unable to exclude the involvement of insulin action^4,5,17^. Inconceivably, little is known about how metabolic surgery affects islet function and glucose homeostasis under physiological conditions. This lack of knowledge tremendously impedes our understanding of mechanisms related to metabolic surgery. To rectify this limitation, we studied the effect of metabolic surgery on glucose homeostasis by eliminating insulin sensitivity and by chemically disrupting islets in healthy rodents. We hypothesized that gastrointestinal rearrangement by metabolic surgery might initiate unique pathways to maintain glucose homeostasis. Thus, we applied RYGB surgery as a tool to study how gastrointestinal intervention affects insulin production and action and the maintenance of glucose homeostasis in a physiological setting, and further validated the findings in the diabetic state.

## Results

### RYGB reduces insulin production but maintains euglycemia

**Fig. 1:**
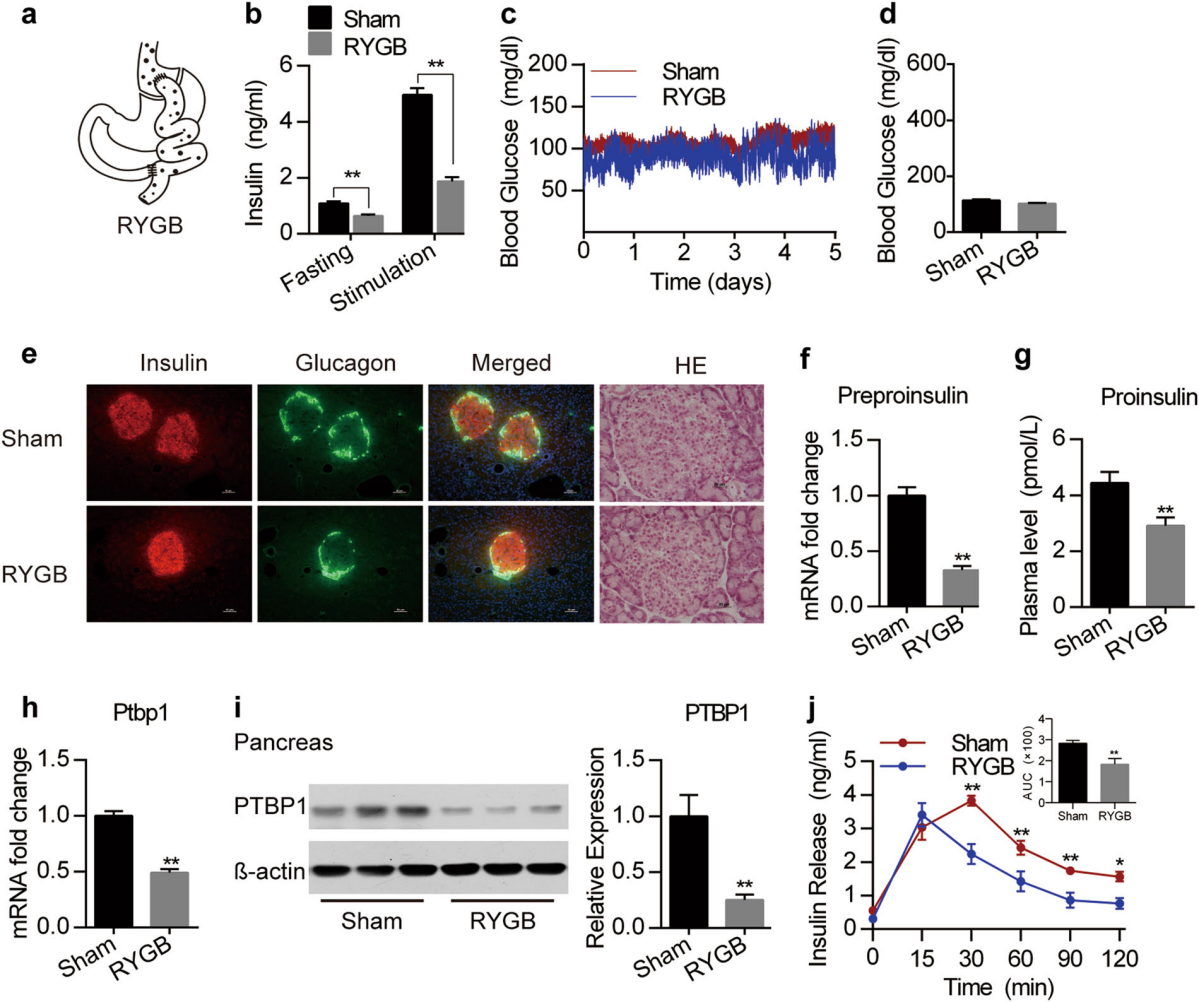
RYGB decreases insulin production and maintains euglycemia. **a** Schematic representation of the RYGB surgery. **b** Plasma insulin levels in fasting and glucose-stimulated states in RYGB and sham rats (*n* = 12). **c** 24 h ambulatory blood glucose levels in sham and RYGB rats (*n* = 6). **d** Mean 24-h ambulatory blood glucose levels in sham and RYGB rats (*n* = 6). **e** Immunofluorescence staining for insulin and glucagon and HE staining of pancreatic islets in sham and RYGB rats. Scale bar represents 50 μm. **f** Relative preproinsulin mRNA expression levels in pancreatic tissues from sham and RYGB rats (*n* = 6). **g** Plasma proinsulin levels in sham and RYGB rats (*n* = 7). **h** Relative Ptbp1 mRNA expression levels in pancreatic tissues from sham and RYGB rats (*n* = 9). **i** Immunoblots of PTBP1 in pancreatic tissues from sham and RYGB rats. Quantitative results are shown on the right (*n* = 6). **j** Plasma insulin levels in sham and RYGB rats and the AUC values for insulin levels after glucose administration (*n* = 8). Values are shown as the mean ± s.e.m. Significance was calculated using two-way ANOVA with Tukey’s post hoc analysis (b), two-tailed unpaired *t*-test (**d**, **e**–**j**), and repeated-measures analysis (**j**). *P < 0.05 and **P < 0.01 compared with the sham group

First, we examined how metabolic surgery affects islet function under physiological conditions. RYGB surgery, which connects a small stomach pouch to the mid-jejunum and bypasses the entire stomach and duodenum, was performed in this study (Fig. 1a). To avoid the interference of insulin defects or resistance, healthy Sprague–Dawley (SD) rats were examined in this study. For dynamic probing of changes in blood glucose levels, we used an updated ambulatory glucose monitoring technique for rats^18^. RYGB predominately reduced basal and glucose-stimulated insulin levels in plasma (Fig. 1b, Supplementary Fig. S1a), but maintained euglycemia in rats (Fig. 1c, d). However, RYGB did not change islet histology; rather, reductions in body weight, food and water intake and urine output were observed in the rats (Fig. 1e and Supplementary Fig. S1b–e). Next, we asked how RYGB reduces plasma insulin levels. Preproinsulin and proinsulin are the precursors of insulin^19,20^. RYGB reduced both pancreatic preproinsulin mRNA and plasma proinsulin levels in rats (Fig. 1f, g). Polypyrimidine tract-binding protein 1 (PTBP1) encodes an insulin granule protein and promotes insulin translation and stabilization^21^. We showed that RYGB remarkably reduced PTBP1 expression at both the mRNA and protein levels (Fig. 1i). Accordingly, RYGB also decreased glucose-induced insulin secretion, as depicted by the area under the curve (AUC) (Fig. 1j). These results suggest that RYGB surgery reduces insulin production and secretion in intact islet conditions but still maintains glucose homeostasis.

### The RYGB-mediated improvement in glucose homeostasis is independent of insulin sensitivity

**Fig. 2:**
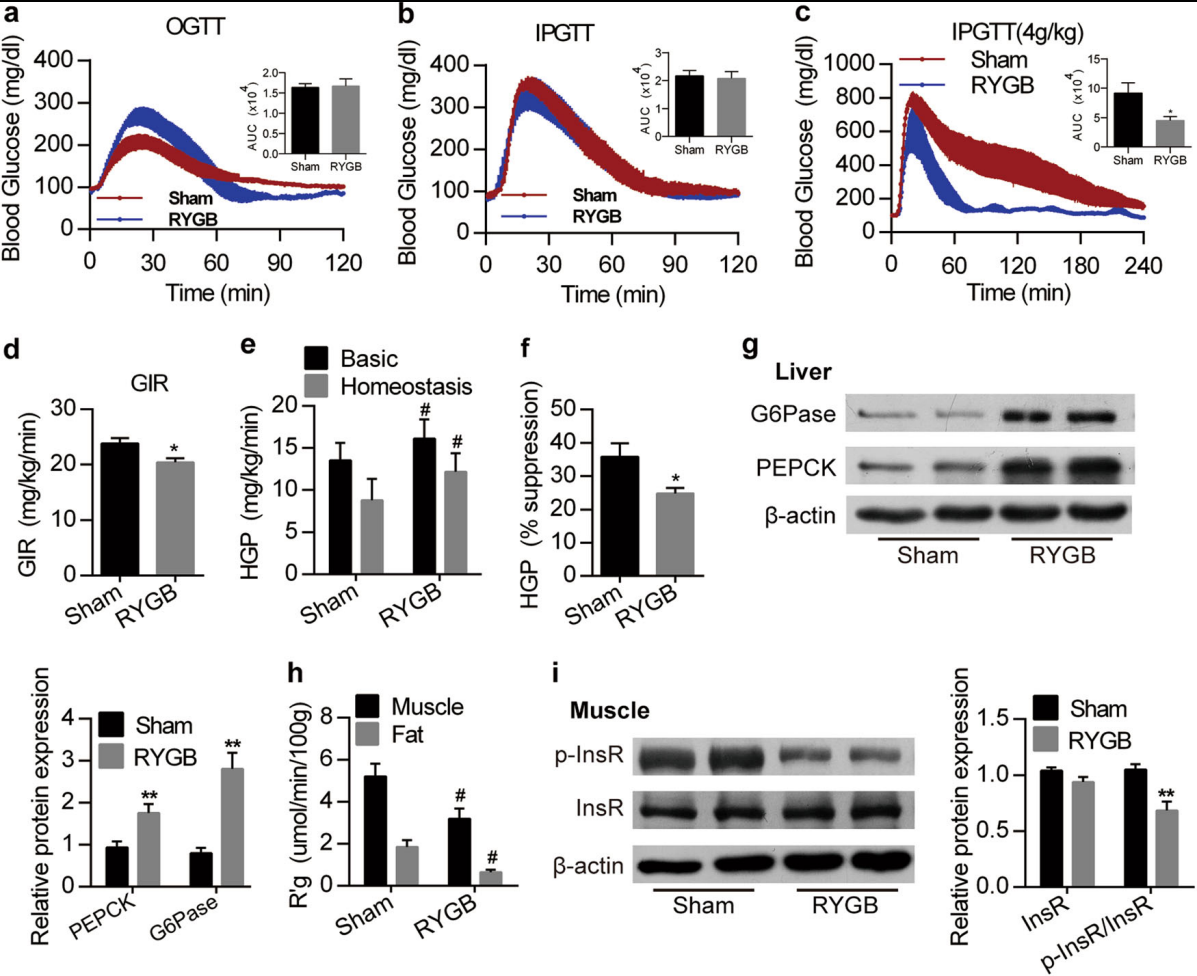
Effect of RYGB on glucose tolerance and insulin sensitivity. **a-c** Results of the OGTT (2 g/kg) (**a**), IPGTT (2 g/kg) (**b**) and IPGTT (4 g/kg) (**c**) for sham and RYGB rats; corresponding AUC values are shown on the right (*n* = 3–6). **d** Glucose infusion rate (GIR) in sham and RYGB rats, determined by hyperinsulinemic-euglycemic clamping in conscious animals (*n* = 9). **e** Hepatic glucose production (HGP) in sham and RYGB rats, evaluated by hyperinsulinemic-euglycemic clamping (*n* = 8). **f** The percentage insulin-mediated reduction in HGP (*n* = 8). **g** Immunoblots of G6Pase, PEPCK and β-actin in the livers of sham and RYGB rats after hyperinsulinemic-euglycemic clamp experiments (*n* = 6). **h** Utilization of glucose in muscle and fat under hyperinsulinemic-euglycemic clamping homeostasis (*n* = 8). **i** Immunoblots of InsR, p-InsR and β-actin 10 min in skeletal muscle after an intraperitoneal injection of insulin into sham and RYGB rats (*n* = 6). Values are shown as the mean ± s.e.m. The significance was calculated using a two-tailed unpaired t-test (**a**–**d**, **f**–**g** and **i**) and two-way ANOVA with Tukey’s post hoc analysis (**e**, **h**). *P < 0.05 and **P < 0.01 compared with the sham group. #*P* < 0.05 compared with the sham group in e and h

Next, we asked whether RYGB affects insulin sensitivity and its related signaling and metabolic pathways. RYGB did not alter oral glucose tolerance (OGT) but did enhance intraperitoneal glucose tolerance (IPGT) after high-concentration glucose infusion in rats (Fig. 2a–c). In this study, we evaluated insulin sensitivity using the hyperinsulinemic-euglycemic clamp test in conscious rats. Insulin is the dominant repressor of hepatic glucose production (HGP)^22,23^. Interestingly, RYGB reduced insulin sensitivity in conscious rats and enhanced HGP under both basal and hyperinsulinemic-euglycemic conditions (Fig. 2d–f), indicating that RYGB attenuated the inhibitory effect of insulin on HGP. In addition, hepatic gluconeogenesis is catalyzed by several key rate-limiting enzymes, such as phosphoenol pyruvate carboxykinase (PEPCK) and glucose-6-phosphatase (G6Pase)^24,25^. Consistent with the enhanced HGP, hepatic PEPCK and G6Pase expression was upregulated under high insulin clamp conditions (Fig. 2f, g). Furthermore, glucose uptake into muscle and adipose tissue was significantly decreased in RYGB rats compared with sham rats, suggesting reduced insulin activity in these insulin-sensitive tissues (Fig. 2h). The glucose uptake by brain or jejunum was increased, whereas it was decreased in heart and not significantly changed in liver and kidney (Supplementary Fig. S2a). Importantly, phosphorylation of the insulin receptor in skeletal muscle was lower in RYGB rats than in sham rats (Fig. 2i). RYGB elevated glucagon-like peptide 1 (GLP-1) levels and decreased dipeptidyl peptidase 4 (DPP-4) and leptin levels in rats without affecting the plasma levels of other gut hormones and adipokines (Supplementary Fig. S2b–i). Taken together, the results indicate that the effect of RYGB on glucose homeostasis is independent of insulin sensitivity; RYGB antagonizes insulin action under physiological conditions.

### Eliminating the stomach and duodenum by RYGB reduces insulin production and restricts energy uptake

**Fig. 3:**
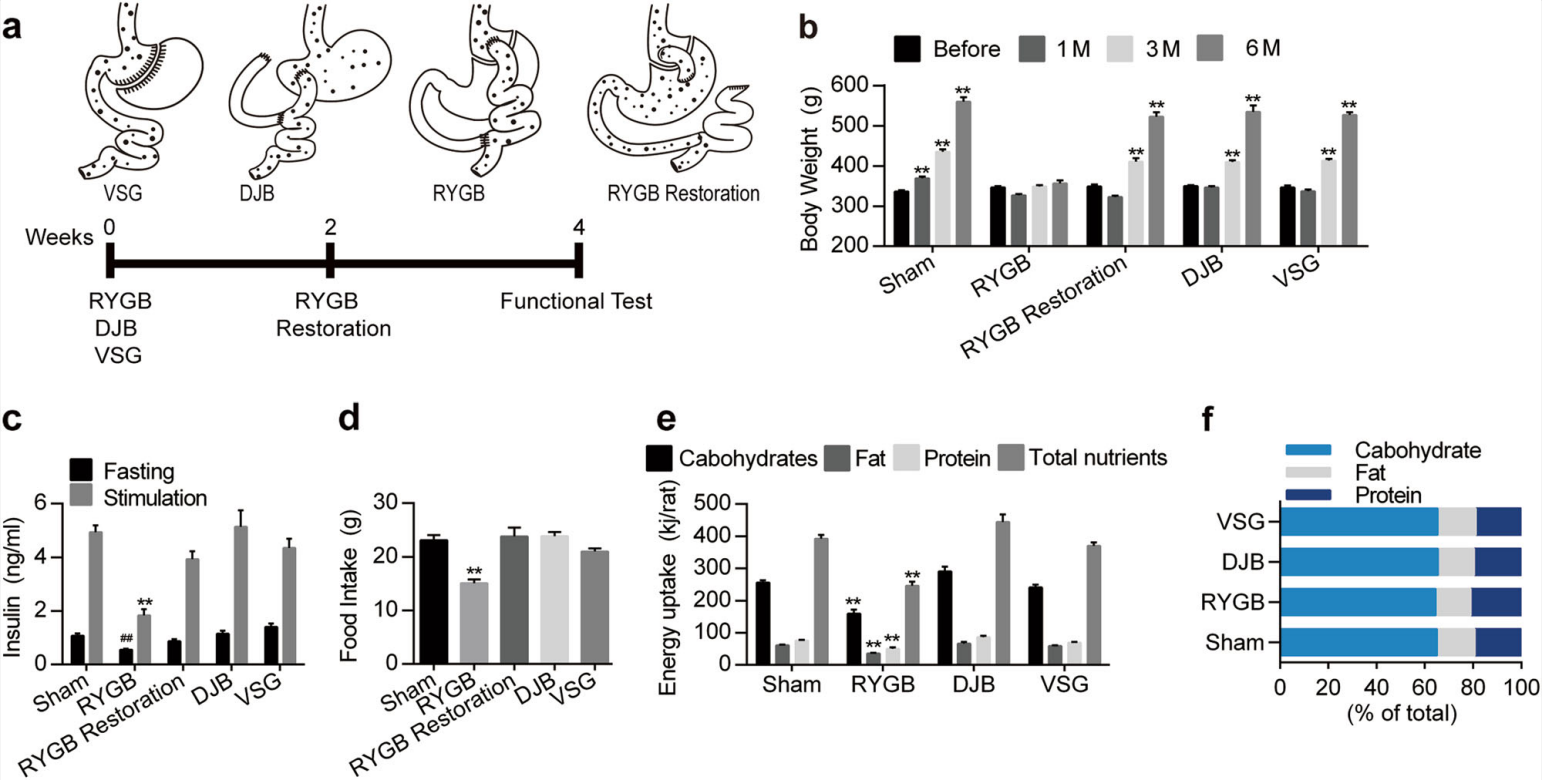
Effects of three metabolic surgeries on plasma insulin and gut energy intake. **a** Schematic of the experimental process and procedures for several metabolic surgeries. **b** Time-dependent body weight changes of rats that underwent the indicated surgeries (*n* = 7). **c** Plasma fasting and glucose-stimulated insulin levels of rats that underwent the indicated surgeries (*n* = 8). **d** Food intake of rats that underwent different surgeries (*n* = 7). **e** Amounts of carbohydrates, fat, protein and total nutrients absorbed by rats that underwent the indicated surgeries, analyzed based on input (food) and output (feces) (*n* = 6). **f** Proportion of absorbed nutrients in rats that underwent the indicated surgeries (*n* = 6). Values are shown as the mean ± s.e.m. Significance was calculated using repeated-measures analysis (**b**) and one-way ANOVA with Tukey’s post hoc analysis (d) and two-way ANOVA with Tukey’s post hoc analysis (**c**, **e**). *P < 0.05 and **P < 0.01 compared with the pre-surgery body weight in **b** and the sham group in **c**–**e**

We further asked which part of the gastrointestinal tract affects glucose homeostasis. We compared the effects of three metabolic surgeries that transform the gastrointestinal tract anatomy in different manners (Fig. 3a). The RYGB procedure is shown in Fig. 1a; the duodenal-jejunal bypass (DJB) procedure bypasses the duodenum and proximal jejunum, while vertical sleeve gastrectomy (VSG) excises most of the stomach^26^. In some rats subjected to RYGB, the original alimentary tract was restored to further confirm the effects of RYGB (Fig. 3a). RYGB, not the other surgical procedures, persistently prevented weight gain and reduced food intake. However, these effects were reversed by alimentary tract restoration (Fig. 3b and d). Importantly, among these surgical procedures, only RYGB significantly reduced plasma insulin levels in rats (Fig. 3c), indicating that the intact stomach and duodenum are critical for insulin production. The intraperitoneal glucose tolerance test (IPGTT), oral glucose tolerance test (OGTT) and intraperitoneal insulin tolerance test (IPITT) results were not affected by the different surgeries (Supplementary Fig. S3a–f). Nutrient digestion and absorption dramatically affect insulin synthesis through nutrient sensing^27,28^. Indeed, analysis of fecal components revealed that RYGB, not the other surgical procedures, reduced energy uptake derived from nutrient absorption by approximately 37% (Fig. 3e). However, none of these surgical procedures altered the proportion of absorbed nutrients in rats (Fig. 3f). We further performed caloric restriction (CR) in rats, whose food intake was 60% of that in sham group. Plasma insulin level was decreased in CR group compared with sham group, but still higher than RYGB group (Supplementary Fig. S3g–i). These results indicate that stomach and duodenum bypass plays a crucial role in the regulation of insulin production through controlling gut energy uptake.

### RYGB shifts the metabolic profile to maintain glucose homeostasis

**Fig. 4:**
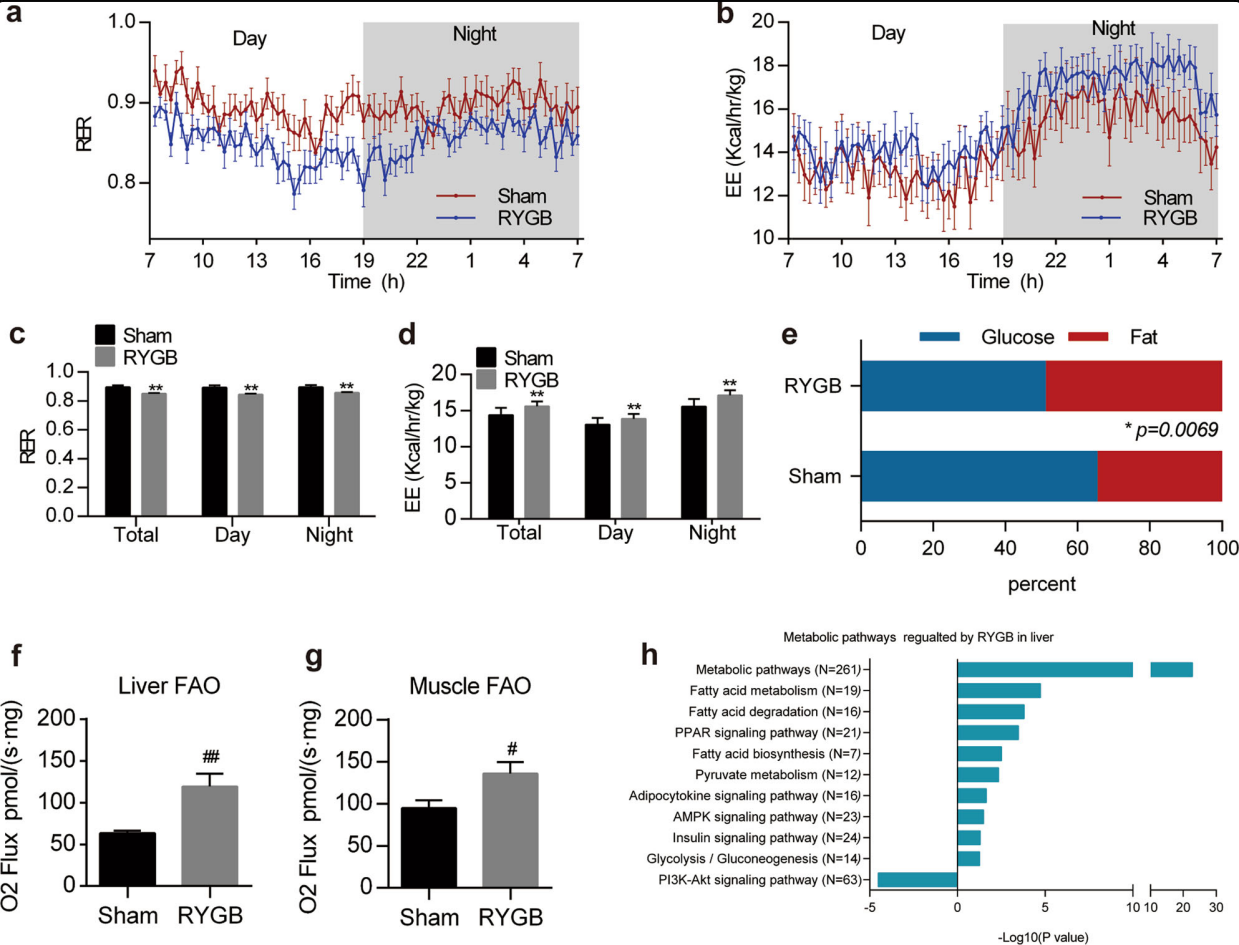
Effect of RYGB on metabolic profiles and energy expenditure. **a–d** RER and EE values of sham and RYGB mice (**a**, **b**) (*n* = 12). Quantitative results are shown in **c, d**. **e** Oxidation ratio of glucose and fat in sham and RYGB rats (*n* = 12). **f**, **g** Mitochondrial fatty acid oxidative activity in the liver and skeletal muscle from sham and RYGB rats (*n* = 8). **h** Enriched metabolic pathways in the rat liver regulated by RYGB (*n* = 3). Values are shown as the mean ± s.e.m. Significance was calculated using two-way ANOVA with Tukey’s post hoc analysis (**c,** **d**) or the two-tailed unpaired t-test (e-g). *P < 0.05 and **P < 0.01 compared with the sham group

**Fig. 5:**
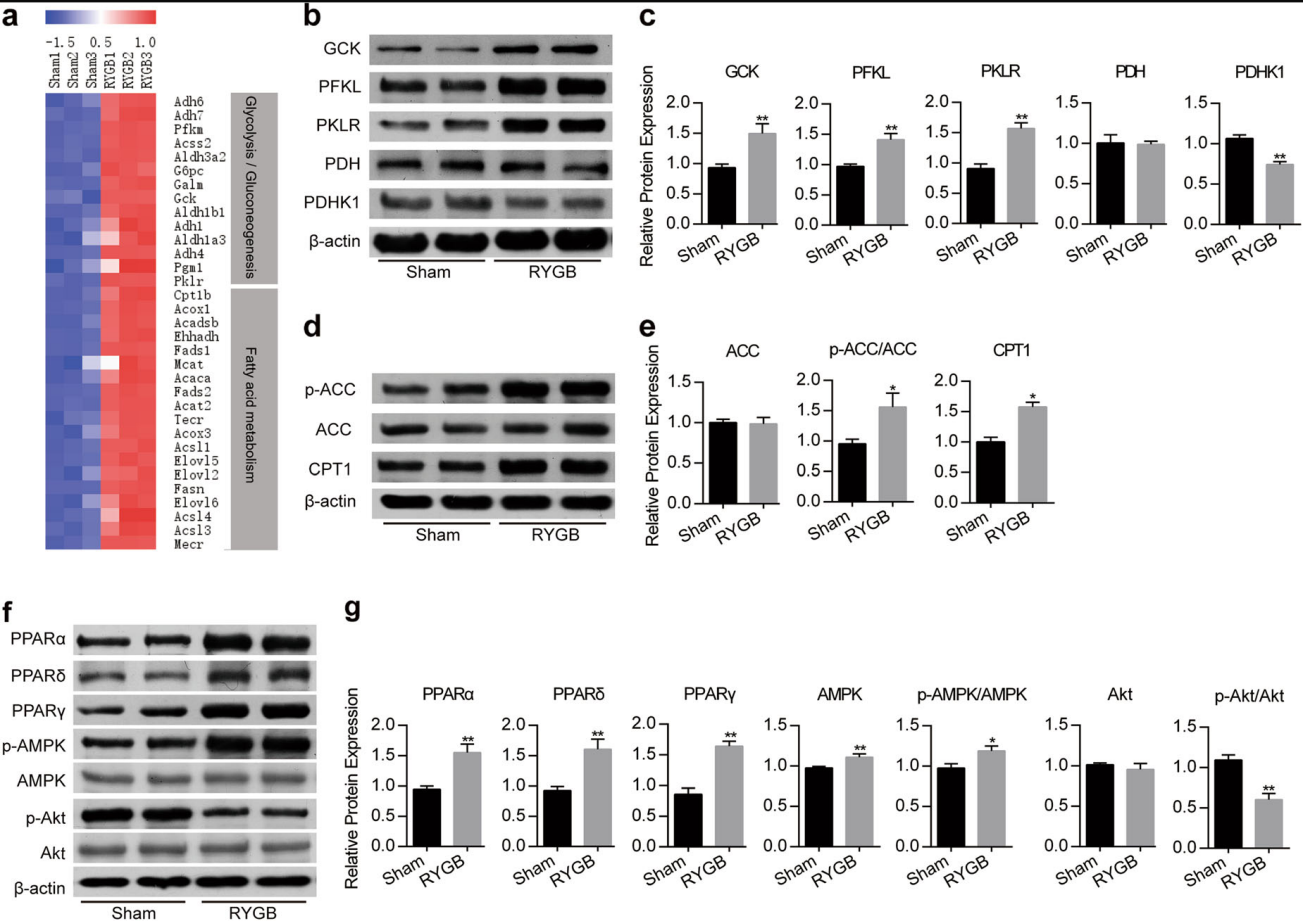
Changes in metabolic-associated genes and enzymes in the liver. **a** Expression levels of enriched glycolysis/gluconeogenesis and fatty acid metabolism pathway genes in the livers of sham and RYGB rats (*n* = 3). **b**, **c** Immunoblots of GCK, PFKL, PKLR, PDH, PDHK1 and β-actin in the liver from sham and RYGB rats (*n* = 6). **d**, **e** Immunoblots of p-ACC, t-ACC, CPT1 and β-actin in the liver from sham and RYGB rats (*n* = 6). **f**, **g** Immunoblots of PPARα, PPARβ, PPARδ, p-AMPK, AMPK, p-Akt, Akt and β-actin in the liver from sham and RYGB rats (*n* = 6). Values are shown as the mean ± s.e.m. Significance was calculated using a two-tailed unpaired t-test (**c**, **e** and **g**). *P < 0.05 and **P < 0.01 compared with the sham group

Then, we asked how RYGB surgery maintains glucose homeostasis in an insulin-paucity state. We showed a clear shift in energy substrate usage from glucose to fatty acids in rodents, as evidenced by a reduced respiratory exchange ratio (RER) at day and night and by increased energy expenditure (EE) in RYGB mice (Fig. 4a–e), suggesting the increasing utilization of glucose and fatty acids as the main energy substrates. Accordingly, octanoyl-fueled mitochondrial oxygen consumption, representing fatty acid oxidation (FAO) in mitochondria, increased in the liver and skeletal muscle in RYGB rats (Fig. 4f–g)^29^. To further explore the metabolic changes after surgery, we performed mRNA microarray experiments on liver, skeletal muscle and adipose tissue. Significantly changed genes (fold change > 2) were identified to perform KEGG pathway analysis (Fig. 4h). In the liver, metabolic-related pathways, such as fatty acid metabolism (including degradation and biosynthesis), glucose metabolism (including pyruvate metabolism and glycolysis/gluconeogenesis) and metabolism-related signaling pathways (including AMPK, peroxisome proliferator activated receptors (PPARs), and adipocytokine), were significantly upregulated. However, the phosphatidylinositol-3 kinase (PI3K)-Akt signaling pathway was downregulated, which corresponded with the result of insulin insensitivity detected by the hyperinsulinemic-euglycemic clamp test in vivo after surgery (Fig. 5f–g). Similarly, PPAR upregulation and PI3K-Akt downregulation were found in skeletal muscle and adipose tissue (Supplementary Fig. S4a, b, Supplementary Fig. S5b–e). Collectively, these results demonstrate that gastrointestinal tract rearrangement alters the manner of energy utilization and activates multiple glucose and fatty acid metabolism-related signaling pathways under insulin-paucity conditions.

### RYGB alters glucose- and fatty acid-related metabolic targets

To verify the mRNA microarray chip results in vivo, some differentially expressed metabolism-associated genes are shown in the heatmap (Fig. 5a, Supplementary Fig. S5a). The enriched genes included key enzymes involved in glucose and fatty acid metabolism. We confirmed the changes in the expression of some of these genes in liver, skeletal muscle and adipose tissue using western blotting. In the rat liver, key genes in glycolysis were enhanced, including glucokinase (GCK), phosphofructokinase liver type (PFKL), and pyruvate kinase isozymes R/L (PKLR). For pyruvate metabolism, pyruvate dehydrogenase kinase (PDHK1) was downregulated (Fig. 5b, c). Regarding fatty acid metabolism, the phosphorylation of acetyl-CoA carboxylase (ACC) and the expression of the FAO key enzyme carnitine palmitoyltransferase-1 (CPT1) were enhanced to promote FAO (Fig. 5d, e). In skeletal muscle, genes involved in glycolysis, such as hexokinase 2 (HK2), 6-phosphofructokinase 1 (PFK1) and pyruvate kinase muscle isozyme 1/2 (PKM1/2), were upregulated after RYGB (Supplementary Fig. S5b, c). In adipose tissue, CPT1 increased after RYGB (Supplementary Fig. S5d, e). These results suggest that RYGB triggers complex changes in the metabolic networks related to glucose and fatty acid metabolism rather than alterations in a single gene or pathway.

### The effect of RYGB on glucose homeostasis has been validated in islet-disrupted rats

**Fig. 6:**
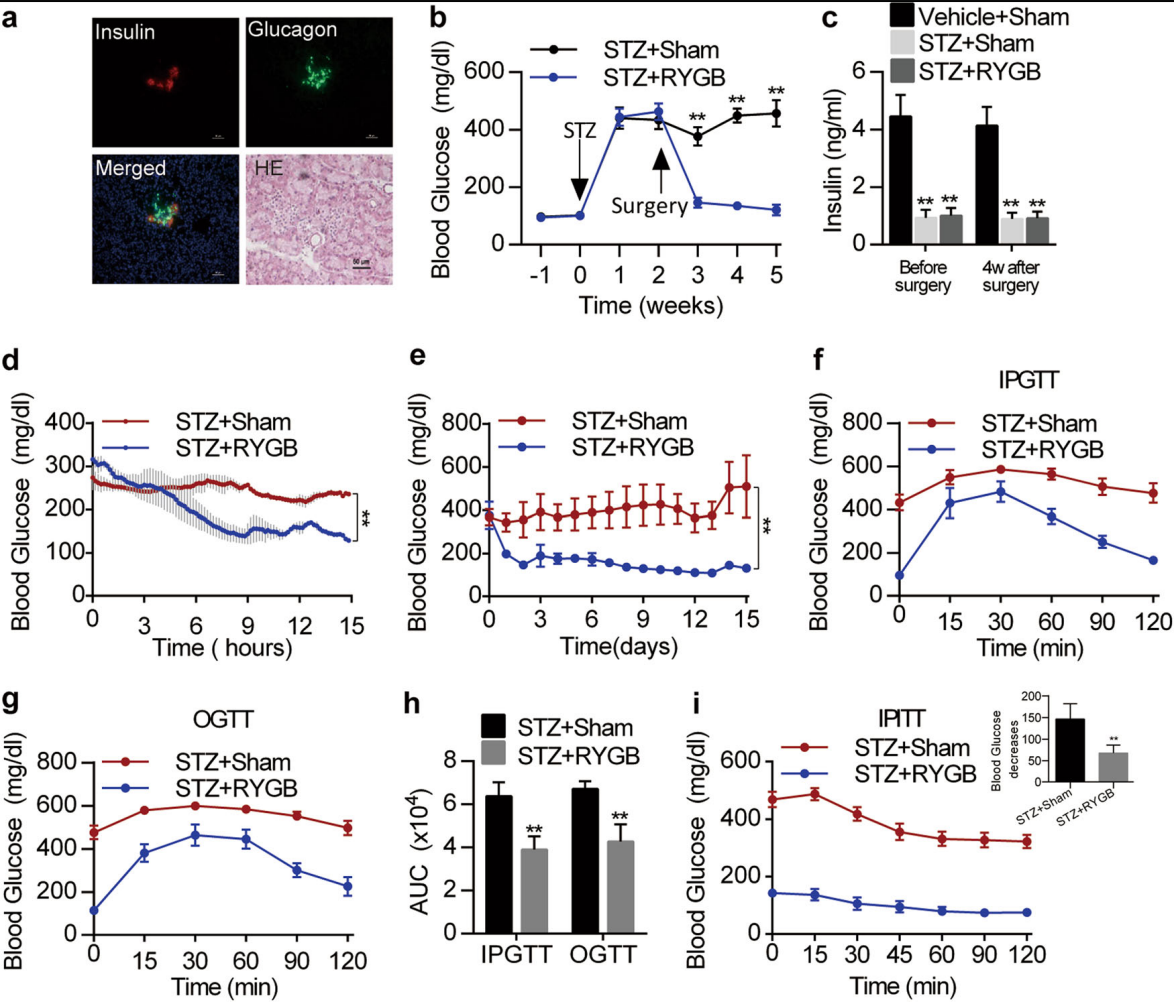
Effect of RYGB on glucose homeostasis in islet-disrupted rats. **a** Immunofluorescence staining for insulin and glucagon and HE staining of pancreatic islets in STZ-treated rats. Scale bar represents 50 μm. **b** Weekly fasting blood glucose levels of the indicated groups from -1 to 5 weeks (*n* = 7). **c** Plasma insulin levels in sham or RYGB treated vehicle or STZ injected rats before and 4 weeks after surgery (*n* = 7). **d** Ambulatory blood glucose levels in sham- or RYGB-treated STZ injected rats within 15 h after surgery (*n* = 2). **e** Mean 24-h ambulatory blood glucose levels sham- or RYGB-treated STZ injected rats within 15 days after surgery (*n* = 2). **f**–**h** Results of the IPGTT (**f**) and OGTT (**g**) for sham- or RYGB-treated STZ injected rats; corresponding AUC values are shown in H (*n* = 4–5). **i** IPITT for sham- or RYGB-treated STZ injected rats. Values are shown as the mean ± s.e.m. Significance was calculated using repeated-measures analysis (**b**) and two-way ANOVA with Tukey’s post hoc analysis (**c**–**e**), and two-tailed unpaired t-test ( **h** and **i**). *P < 0.05 and **P < 0.01 compared with the surgery group in **b** and **d**–**e**, compared with the sham group in **h** and compared with the vehicle + sham group in **c**

We have demonstrated that RYGB can maintain glucose homeostasis in an insulin-paucity state under physiological conditions. We reasoned that this effect can be validated during insulin deficiency. Thus, we disrupted rat islets through a single bolus peritoneal injection of streptozocin (STZ). STZ administration severely destroyed islets (Fig. 6a) and tremendously decreased plasma insulin levels in SD rats (Fig. 6c). STZ-treated rats displayed typical features of diabetes (Supplementary Fig. S6a–d). However, RYGB restored robust hyperglycemia to euglycemia within one week in STZ-treated rats compared with sham surgery (Fig. 6b). Furthermore, the insulin-deficient state was not altered in STZ-treated rats before and after surgery (Fig. 6c). To further examine the changes in blood glucose, a glucose telemetric transmitter was surgically implanted into the distal portion of the descending aorta. The results further validated that RYGB persistently maintained long-term euglycemia in rats with islet disruption (Fig. 6d, e). In addition, IPGT and OGT were significantly improved in STZ-treated rats after surgery (Fig. 6f–h). However, insulin sensitivity, which was evaluated by IPITT, was declined in STZ-treated rats after surgery (Fig. 6i). These results indicate that RYGB can rapidly antagonize hyperglycemia and maintain glucose homeostasis, even in conditions of severe islet disruption.

### The ability of RYGB to remit hyperglycemia is independent of islet state in patients with type 2 diabetes

**Fig. 7:**
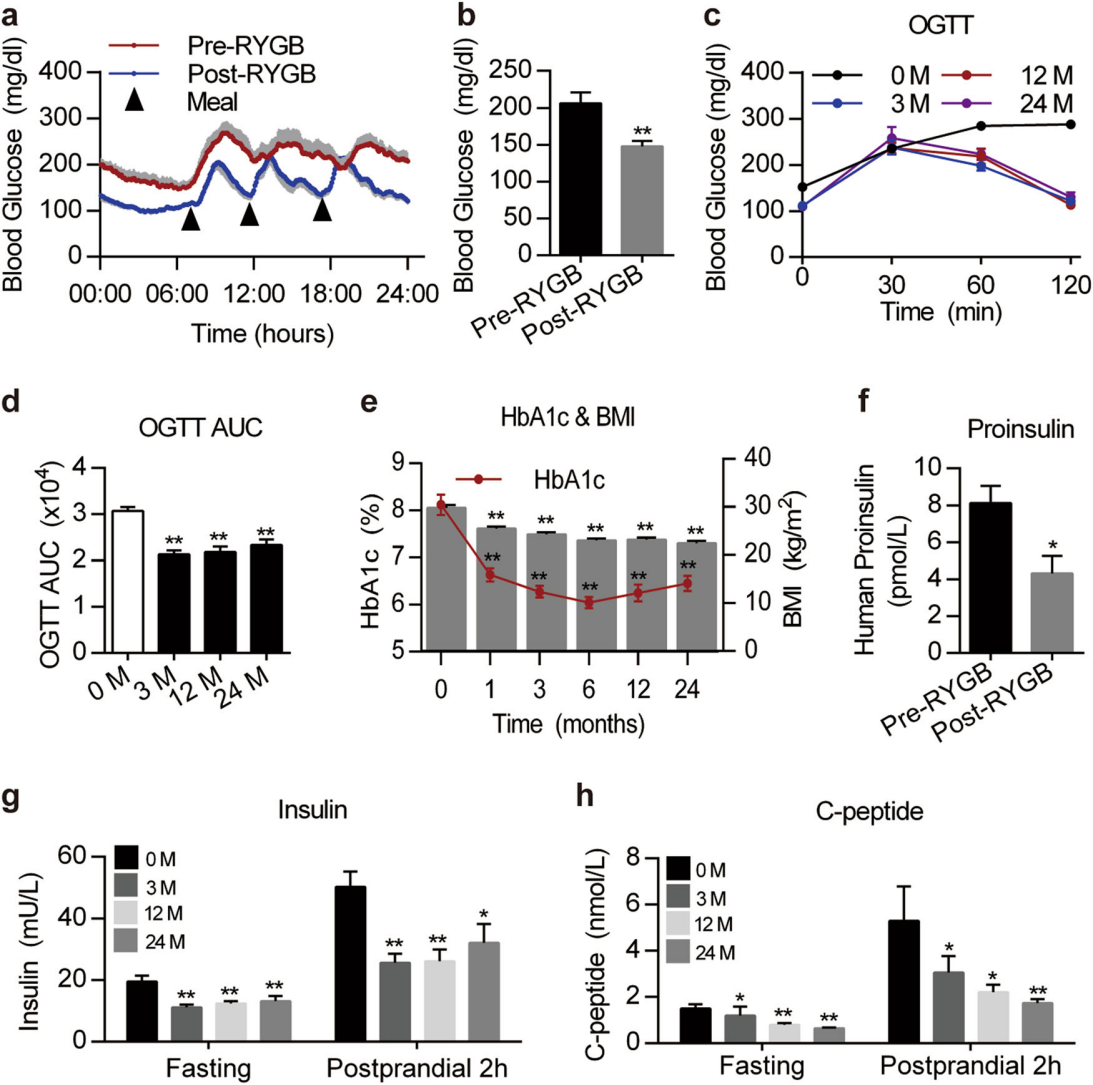
RYGB remits hyperglycemia in patients with type 2 diabetes. **a**, **b** 24-h dynamic and mean blood glucose values in patients pre- and post-RYGB surgery (*n* = 9 pre-RYGB and 15 post-RYGB). **c**, **d** OGTT results for patients at 0, 3, 12 and 24 months after surgery (*n* = 25–83). **e** Hemoglobin A1c (HbA1c) levels and body mass index (BMI) at different time points for T2DM patients who underwent RYGB surgery (*n* = 23–87). **f** Serum proinsulin levels of patients before and after surgery (*n* = 9–12). **g** Plasma insulin levels and AUC values of the insulin release test at different time points for T2DM patients who underwent RYGB surgery (0 M (pre-RYGB); 3 M (3 months); 12 M (12 months); 24 M (24 months), *n* = 25–70). **h** Serum C-peptide levels of T2DM patients at the indicated times before and after RYGB upon glucose stimulation for 2 h (*n* = 24–78). Values are shown as the mean ± s.e.m. The significance was calculated using a two-tailed unpaired *t*-test (**b**), Mann–Whitney nonparametric U test (**f**), and repeated-measures analysis (**d**, **g**–**h**). *P < 0.05 and **P < 0.01 compared with the pre-RYGB values

Currently, RYGB is not recommended for patients with type 2 diabetes with severe islet lesions^6^. However, the beneficial effect of RYGB in patients with type 1 diabetes has also been reported^30^. In this study, we examined the relationship between islet function and blood glucose change in patients with type 2 diabetes before and after RYGB. General characteristics of the patients are included in the supplemental information (Supplementary Table S1). We showed that the average ambulatory and fasting blood glucose levels were significantly reduced in patients with diabetes after surgery (Fig. 7a, b). Compared with the OGT test results before surgery, those for patients with diabetes after surgery were improved and maintained over long term (Fig. 7c, d). RYGB led to a persistent decline in HbA1c and body mass index (BMI) in patients with diabetes compared with the pre-surgery values (Fig. 7e). RYGB decreased the plasma levels of proinsulin, insulin and C-peptide in patients with diabetes (Fig. 7f–h). Currently, most clinical studies have shown that RYGB decreases plasma insulin levels in obese patients with type 2 diabetes^31–33^. Our clinical evidence further indicates that the euglycemic status maintained by RYGB might be independent of insulin and islet state in patients with diabetes.

## Discussion

**Fig. 8:**
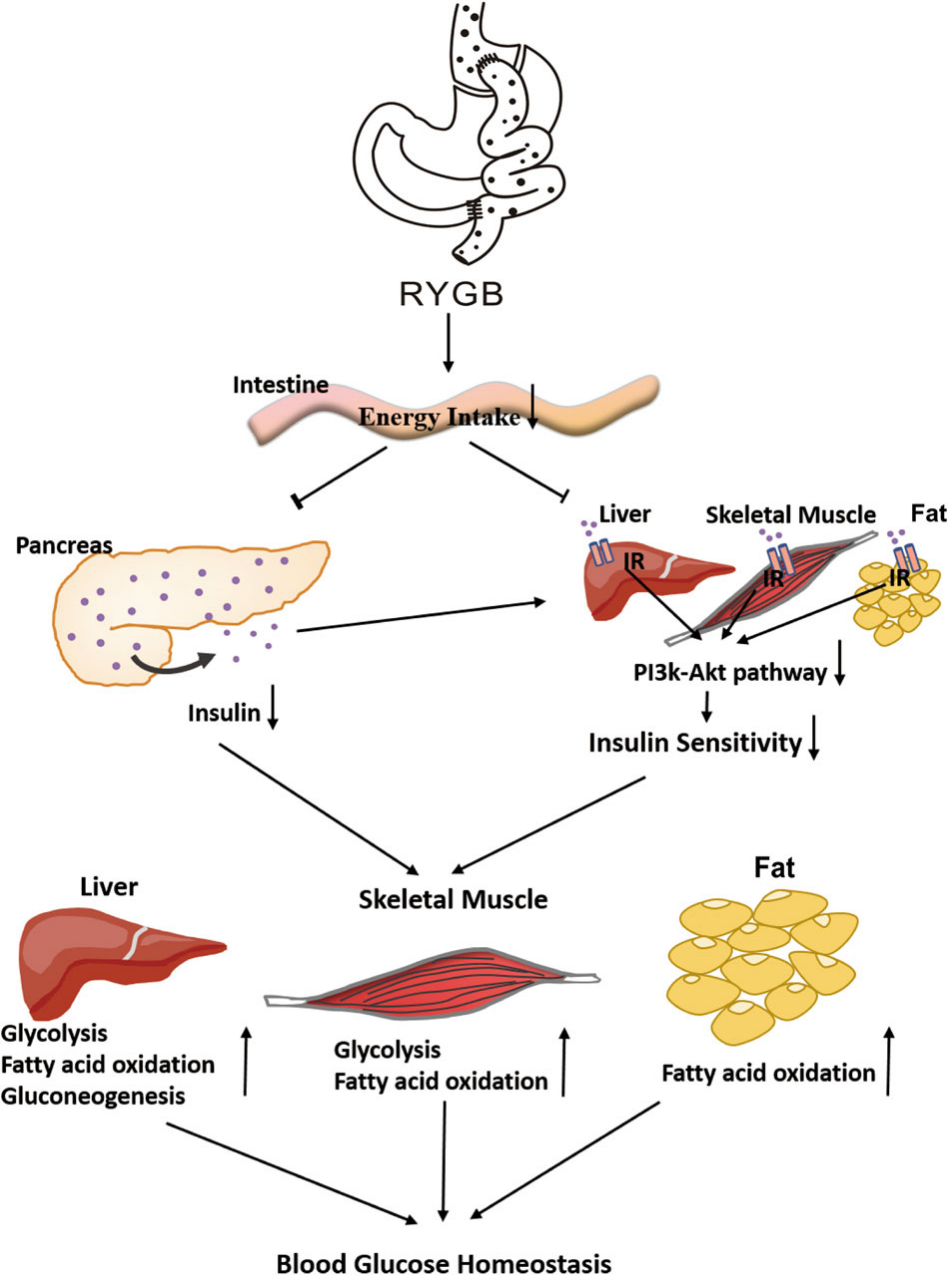
Schematic of the mechanism of non-insulin determinant metabolic pathways. Excluding the stomach and duodenum by RYGB in rats largely reduces energy intake, which further decreases insulin production and inhibits the insulin signaling pathway in the liver, skeletal muscle and fat. Thus, insulin-independent metabolic pathways are triggered, including enhanced glycolysis, fatty acid oxidation and gluconeogenesis. Consequently, glucose homeostasis is initiated by non-insulin determinant metabolic pathways. IR insulin receptor

In this study, we utilized RYGB metabolic surgery as a tool to identify the non-insulin determinate mechanism that is crucial for maintaining glucose homeostasis. Importantly, we reveal that gastrointestinal tract rearrangement by RYGB reduces both insulin production and action but persistently maintains euglycemia in healthy rats. The mechanistic evidence suggests that RYGB antagonizes pancreatic preproinsulin expression and the insulin signaling pathway in several insulin-sensitive tissues. Due to the restriction of gut energy uptake, RYGB predominately shifts the metabolic profile from glucose utilization to FAO, enhances EE and triggers multiple metabolic pathways. Importantly, these unique effects of RYGB can be extended to rats with islet disruption and patients with type 2 diabetes. Based on our experimental and clinical results, we propose that excluding the stomach and duodenum by RYGB in rats largely reduces energy intake, which decreases insulin production and triggers insulin-independent metabolic pathways. Consequently, glucose homeostasis is maintained by non-insulin-dependent mechanisms (Fig. 8).

The liver, skeletal muscle and fat harmonize to maintain whole-body glucose homeostasis^3^. Ingested glucose is absorbed from the gastrointestinal tract into the portal vein and liver. There, the liver eliminates and stores a large fraction of ingested glucose while decreasing the amount of hepatic glucose entering the circulation^34^. Insulin is a depressor of HGP, whereas hepatic glucose uptake is less sensitive to insulin^22^. Conversely, when glucose is no longer absorbed from the gastrointestinal tract, HGP will increase to avoid hypoglycemia through declining insulin secretion from pancreatic islets. In this study, RYGB resulted in a 37% reduction of ingested nutrients by eliminating the stomach and duodenum. This compulsory restriction of gut energy uptake inhibits insulin synthesis because starvation or caloric restriction significantly decreases plasma insulin levels in humans^35^. In addition, insulin is derived from preproinsulin and proinsulin^19,20^. PTBP1 binds mRNAs that encode insulin granule proteins and promotes insulin translation and stabilization^21^. We found that RYGB directly inhibited pancreatic preproinsulin and PTBP1 expression and plasma proinsulin levels. However, the effect of RYGB on the structure and function of secretory granules in pancreatic islets worthy further investigates through electron microscope analysis. It is reported that insulin sensitivity also contributes to the maintenance of glucose homeostasis^36^. Unexpectedly, RYGB decreased, not increased, insulin sensitivity in this study. The following evidence supports this point: 1. the hyperinsulinemic-euglycemic clamp results showed a decrease in insulin sensitivity in conscious rats; 2. phosphorylation of the insulin receptor and flux through the PI3K/Akt signaling pathway were reduced in the liver, skeletal muscle and fat; 3. HGP was enhanced under both basal and hyperinsulinemic-euglycemic conditions, indicating weaker insulin action; and 4. decreased glucose uptake was observed in skeletal muscle and adipose tissue. From an evolutionary view, mammalian survival has adapted to insulin-independent circumstances because of food scarcity. In times of famine, starving people rarely suffer from obesity and type 2 diabetes despite insufficient energy intake diminishes insulin production and the energy expenditure to avert hypoglycemia^5,37^. In fact, the effect of RYGB somewhat resembles a moderate famine state by placing a compulsory limit on gut energy intake. Taken together, our results demonstrate that the effects of RYGB on glucose homeostasis are independent of insulin action. Furthermore, these effects can be validated in rodents and patients with diabetes regardless of their islet status.

The next critical question is how glucose homeostasis is maintained under insulin deficiency and insensitivity. Although muscle and adipose tissue are insulin insensitive and uptake less glucose in rats after RYGB, glucose uptake is enhanced in brain and jejunum in this study. It is well known that glucose is a critical energy substrate required for brain functioning^38^, and the intestine exhibited the highest rate of glucose uptake and became a major tissue for glucose disposal after RYGB^11^. Thus, the decreased glucose absorption in metabolic organs, but enhanced glucose utilization of vital organs might contribute to the maintenance of glucose homeostasis after RYGB.

The binding of insulin to its receptors activates the canonical insulin receptor substrate/PI3K pathway, which potently inhibits both glycogenolysis and gluconeogenesis^2^. Gluconeogenesis is catalyzed by several key enzymes, such PEPCK and G6Pase^24,25^. Glycolysis is catalyzed by PFK, PK, and HK, among others^39^. We showed that RYGB markedly increased the expression of these key enzymes in the rat liver, skeletal muscle and adipose tissue. In addition, this study clearly showed a shift in energy substrate usage from glucose to fatty acid metabolism after surgery. RYGB enhanced fatty acid degradation and promoted fatty acid utilization. These alterations are involved in changes in ACC, CPT1. Importantly, RYGB also impacted a plethora of crucial signaling molecules that regulate glucose and fatty acid metabolism, such as AMPK and PPARs. Some of these molecules are targets of hypoglycemic drugs, such as the AMPK activator metformin, GLP-1 agonists, adiponectin, and PPAR activators such as those in the glitazone family^40,41^. Although incretin hormones play an important role for glucose homeostasis, and plasma GLP-1 level elevated after RYGB in this study, previous study shows that GLP-1 is not the cause of the amelioration of hyperglycemia after RYGB^42^. Thus, no single drug or drug combination has been able to completely normalize blood glucose, as compared with RYGB^9,32,43^. Our study indicates that a complex metabolic network, not a single target, is triggered by RYGB, which is responsible for the regulation of glucose homeostasis.

It was astonishing that RYGB led to not only weight loss but also glucose control and end-organ health^9^. Clinical studies have demonstrated that metabolic surgery also remits cardiometabolic complications, dementia, and cancer beyond the remission of hyperglycemia^8,9,44^. Hyperinsulinemia is regarded as the root cause of these noncommunicable diseases^7,45^. In modern society, excessive energy uptake and lower energy expenditure lead to hyperinsulinemia and fat accumulation in humans^5,46^. Persistent hyperinsulinemia leads to cellular and target organ dysfunction^46,47^. Therefore, caloric restriction and physical exercise are essential to maintain energy balance by antagonizing the action of hyperinsulinemia^48–50^. Although lifestyle changes remarkably improve insulin sensitivity and glucose homeostasis, these behavioral interventions suffer from poor compliance and are insufficient to overcome overt obesity and diabetes^48,49^. These findings suggest that the principle mechanisms of RYGB are distinguished from those of lifestyle changes. In addition, our microarray chip analysis showed that RYGB impacted genes involved in inflammation, oxidative stress, cell growth, immunity, etc. These factors are closely associated with the development of cardiometabolic diseases, cancer, and brain disorders^51^.

In summary, a strong non-insulin-dependent mechanism maintains glucose homeostasis and energy balance in physiological states. Disrupting this mechanism likely leads to disorders characterized by energy metabolism dysregulation. Based on the mechanism of action of RYGB, the development of a noninvasive intervention involving the gastrointestinal tract is a promising therapeutic route to combat these disorders.

## Materials and methods

### Animals and rodent diabetes model

The animal experiments and animal care were performed in compliance with and were approved by the ethics committee of the third affiliated hospital to the Third Military Medical University. Non-obese male Sprague Dawley (SD) rats, C57BL/6 J mice were used in this study. Rats and mice were housed under a 12-hour day-night cycle and had free access to food and water. Rats were randomly assigned to the sham-treated or metabolic surgery groups. The protocol is provided in the text. After an overnight fast, SD rats were intraperitoneally injected with vehicle (0.1 M sodium citrate, pH 4.5) or 45 mg/kg STZ (Sigma-Aldrich Co LLC, St. Louis, MO) according to the protocol from the Animal Models of Diabetes Complications Consortium (AMDCC). After three days, rats were described as diabetic if their fasting blood glucose levels were greater than 300 mg/dL (16.7 mmol/L) for three consecutive days^52–54^.

### Oral glucose tolerance test (OGTT) and intraperitoneal glucose tolerance tests (IPGTT)

Animals were fasted overnight before all the tests. After fasting, animals were given the glucose solution (2 g glucose/kg body weight) orally for OGTT, or glucose solution (2 g glucose/kg body weight or 4 g glucose/kg body weight) injected intraperitoneally for IPGTT^55,56^. OGTT and IPGTT were performed in separate days. Blood glucose were monitored by the glucose telemetric transmitter before and after the test, or using the Nova StatStrip Xpress glucometer (Nova Biomedical, Waltham, MA) by cutting the tail and gently massaging blood onto a glucose test strip.

### Surgery and post-surgery procedures

After an overnight fast, animals were deeply anesthetized with isoflurane (4% induction and 1.5% maintenance; VIP-3000 Vaporizer; Matrix, Orchard Park, NY), and the abdominal organs were exposed by laparotomy. Animals were fasted for 12 h after surgery. After evaluating the health status and behavior of each animal, a limited liquid diet was reintroduced 12–72 h after the operation. Three days after surgery, normal chow was provided ad libitum to the animals^11,44^. Animals used in this study underwent operations in different cohorts, and STZ was injected 2 weeks before surgery^57^.

### Rat RYGB

The total length of the small intestine was measured, the ligament of Treitz was identified, and the small intestine was divided at the appropriate distance (8–10 cm) downstream of this ligament^11^. A gastrojejunostomy and an end-to-side jejuno-jejunostomy (18–20 cm from the ligament of Treitz) were created with a 6–0 silk interrupted suture^57^. A gastric pouch consisting of 10% of the total stomach volume was created using scissors, and the distal cut end of the stomach was sutured with 6–0 silk. The intestine remained hydrated during this procedure. The gastric artery was preserved, and only the small vascular branches were cauterized with electrocoagulation. The laparotomy was closed with a 4–0 Prolene suture in two layers.

### Mouse RYGB

The upper gastrointestinal tract was exposed, and the length of the small intestine was measured. The intestine was cut at a distance of 10–15% of the intestinal length from the pylorus. The stomach was gently dissected from the spleen and liver by using cotton swabs, and the gastro-esophageal junction was fully externalized. The left gastric vessels and vagal fibers were removed to create a small pouch (approximately 2% of the stomach capacity). The stomach remnant was closed using a continuous stitch with an 8–0 Prolene suture (Ethicon US, LLC, USA). The stomach pouch was then anastomosed with a distal jejunum, and a jejuno-jejunostomy was performed using an interrupted stitch with an 8–0 Prolene suture at a distance of 10–15% of the intestinal length from the gastrojejunostomy. Warm saline was regularly added to wet the tissue. The abdominal muscle and skin were closed using an interrupted stitch with a 6–0 silk suture^26^.

### Vertical sleeve gastrectomy (VSG)

The stomach was isolated outside the abdominal cavity and placed on warm saline-soaked gauze pads. It was then freed from the spleen and liver, and the lateral 80% of the stomach was cut, leaving a tubular gastric remnant (closed using an interrupted stitch with a 6–0 silk suture) while keeping the proximal and distal ends intact. Finally, the abdominal wall was closed in layers using an interrupted stitch with a 4–0 silk suture^26^.

### Duodenal-jejunal bypass (DJB)

The stomach was fully externalized with a VSG surgical procedure, and the jejunum was transected using the previously described rat RYGB procedure. The pyloric sphincter was ligated with a 4–0 silk suture. An incision was created on the lateral wall of the stomach, and the distal jejunum was anastomosed to the stomach. A jejuno-jejunostomy was performed with an RYGB procedure. The abdominal wall was closed in layers using an interrupted stitch with a 4–0 silk suture^10^.

### Restoration of the gastroduodenal passage after RYGB surgery

A loop of the jejunum (1 cm) was isolated and transposed between the oesophagus and the stomach to restore the gastroduodenal passage of nutrients after RYGB surgery^58^. After the operation, nutrients flowed from the esophagus through the jejunal loop to the stomach in a latero-terminal fashion involving the duodenum, jejunum, and remaining intestinal tract. Anastomoses were performed with a 6–0 silk interrupted suture^11^.

### Sham operation

In all experiments, animals undergoing a sham operation were used as controls. The sham operation was consisted of laparotomy, jejunal transection, and sutured; and was performed in animals that were matched (age- and weight-) to rats underwent RYGB or restoration.

### Continuous blood glucose monitoring in rats

Glucose telemetric transmitters (HD-XG transmitter; Data Sciences International, Saint Paul, MN) were surgically implanted in SD rats after metabolic surgery and before the STZ injection, according to a previously reported protocol^18,59^. The catheter portion of the implant was placed into the distal portion of the descending aorta. After rats recovered from surgery for several days, the blood glucose levels in conscious and unrestrained rats were measured by telemetry until the end of the study. We collected data for 10 s every minute and used the mean values for the statistical analysis.

### Hyperinsulinemic-euglycaemic clamp

The test was performed as previously described, and insulin sensitivity was represented by the amount of glucose infused during the last 60 min of clamping. In detail, two catheters were separately introduced into the carotid artery and right jugular vein. Briefly, a primed continuous infusion of HPLC–purified [3-H3] glucose (Amersham, Los Angeles, CA; 6 mCi bolus, 0.2 mCi/min) through the carotid artery catheter was initiated at 0 min and maintained throughout the study. The hyperinsulinemic-euglycemic clamp was performed, insulin (6 mU/kg/min) was continuously infused, and a variable infusion of 25% glucose was started and adjusted every 5 min to maintain the plasma glucose concentration at 6 mmol. GIR = Y × 25/(6 × W) (Y: glucose infusion rate, ml/h; W: body weight, kg). Blood samples were collected from the jugular vein catheter at 0, 120, 200, 220, 230, and 240 min and used to determine insulin levels. To ascertain insulin-mediated glucose uptake in individual tissues, 2-deoxy-D-[H3] glucose (2-DG) (Amersham; 30 mCi bolus) was administered 45 min before the end of the clamp studies. Extra blood samples (100 µL) were taken at 2, 5, 10, 15, 20, 30 and 45 min after the infusion to determine tracer disappearance. At the end of the clamp, the rats were anesthetized, and tissue samples were frozen in liquid nitrogen and stored at −80 °C for subsequent analysis. Insulin sensitivity was computed with previously described formulas^60^.

### Isolation of skeletal muscle and liver mitochondria

The rats were sacrificed, and their musculi soleus tissues were promptly removed, washed with phosphate-buffered saline, and weighed. The tendon was removed. Each musculi soleus was placed in ice-cold buffer (180 mM KCl, 10 mM Tris, and 0.5 mM Na_2_EDTA, pH 7.4), cut into tiny pieces, and further homogenized in buffer containing 180 mM KCl, 10 mM Tris, 0.5 mM Na_2_EDTA, and 1 g/L BSA, pH 7.4, by using a Dounce homogenizer. Muscle mitochondria were isolated by differential centrifugation. Mitochondria were weighed and suspended in MiR06 solution. The remaining sample was stored at −80 °C for subsequent analysis^61^.

### High-resolution respirometry

The mitochondrial respiration rate was measured by using an Oxygraph-2k (Oroboros, Innsbruck, Austria) with two chambers at 37 °C, and 1.0 mg of mitochondria was added into each high-resolution respirometry chamber containing 2 mL of MiR06 solution. The Datlab software package (Oroboros Instruments) was used for data acquisition and analysis. The substrate–uncoupler–inhibitor titration (SUIT) reference protocol used to evaluate mitochondrial function included ADP (450 μM), Mitochondria (Mt), Oct (0.5 mM), Malate (0.05 mM, 0.1 mM, and 2 mM), cytochrome c (10 μM), a titration of the uncoupler FCCP (injected stepwise up to 1–1.5 μM), rotenone (0.5 μM), and myxothiazol (0.5 μM). The ADP was added before Mt to accelerate the depletion of endogenous substrates. Then, the fatty acid oxidation (FAO) rate was determined by using Oct and Malate. Cytochrome c was used to confirm the integrity of the outer mitochondrial membrane. FCCP was titrated to evaluate the maximal electron transfer system (ETS) capacity. Residual oxygen consumption (ROX) was determined by adding rotenone and myxothiazol.

### Energy expenditure and Respiratory Exchange Ratio

To investigate the effect of RYGB surgery on mouse energy expenditure (EE) and respiratory exchange ratio (RER), the Comprehensive Laboratory Animal Monitoring System (CLAMS; Columbus Instruments, OH, USA) was used to monitor the oxygen consumption and respiratory exchange ratio (RER) of mice. Mice were weighted and then housed individually in monitoring cages for 48 h and the room temperature was maintained in 22 °C under a 12-hour/12-hour day/night cycle. Mice were acclimatized to the cage for 24 h before each trial. Data was analyzed by the suited Oxymax software package (Columbus Instruments). The contents of oxygen (O2) and carbon dioxide (CO2) in the air sampled from the cage was determined by the separated sensor (Columbus Instruments). Carbon dioxide output (VCO2) and oxygen uptake (VO2) was counted between each interval. Then RER was calculated according to the equation RER = VCO2 / VO2. Heat production was calculated according to the equation: EE = (3.815 + 1.232 × RER) × *V*O2 × 0.001 (kcal/ [kg × h])^62^.

### Metabolic assays and ELISA kits

For insulin, glucagon, DPP-4, Ghrelin, Leptin, Gastrin, and FGF-21 assays, blood was collected into heparinized tubes on ice. For GIP and GLP-1(7–36) assays, blood was collected into tubes, on ice, containing EDTA and dipeptidyl peptidase IV (DPP-IV) inhibitor (Millipore, Billerica, MA, USA)^10^. Plasma was obtained by centrifugation of heparinized blood at 4 °C for 20 min at 1,200 g and subjected to enzyme immunoassays. The plasma glucagon, GLP-1 (7–36), GIP, Ghrelin, Leptin (Millipore, Billerica, MA, USA) and DPP-4 Gastrin, and FGF-21 assays were performed using commercialized kits according to manufacturer’s instructions. The plasma insulin levels were determined by ELISA kits and radioimmunoassay (ELISA, Mercodia, Uppsala, Sweden and RIA, Millipore, Billerica, MA, USA), respectively. All the samples were performed in bipartites.GLP-1 levels were measured 30 min after a meal of glucose solution (2 g/kg body weight).

### Fecal analysis

24-hours fecal pellets from individual rat were collected to have sufficient material for analysis. Fecal total protein concentrations were quantified using Kjeldahl method^63^, total fat were extracted and measured by the procedure of Soxhlet extraction. Total carbohydrate and totalcalories were estimated as follows:

Total carbohydrate (%) = 100-(Ash + Moisture + Protein + Fat) %

Total calories (kj) = Carbohydrate × 17 + Protein × 17 + Fat × 37

Results were then calibrated with the weight of the sample.

### Histology and Immunofluorescent staining

Whole pancreas was embedded in Tissue Tek (Sakura Fneteci), frozen, cut into sections (8 um). Histology slides containing sections from the pancreas were fixed with 4%paraformaldehyde, permeabilized with 0.01% Triton X-100.For histology, the slides from each group were stained with hematoxylin and eosin. For Immunofluorescent staining, nonspecific binding sites were blocked in 10% bovine serum in PBS at room temperature for 30 min. The slides were then incubated at 4 °C overnight with diluted primary antibodies and with Alexa Fluor labeled secondary antibodies at 37 °C for 60 mins in the dark followed by DAPI for 30 mins at room temperature in the dark. The slides were then rinsed with PBS and evaluated under a fluorescence microscope. The primary antibodies used were anti-insulin antibody (ab6995, Abcam), anti-glucagon antibody (ab8055, Abcam). The images were collected using a Nikon TE2000-U inverted fluorescence microscope (Nikon Co., Tokyo, Japan).

### Western blot

Protein was extracted from the frozen tissues of animals, including islet, liver, skeletal muscle and white adipose tissue. Protein concentrations were determined by BCA method. Equal amounts of protein (20 μg per lane) were separated by SDS-PAGE and transferred onto polyvinylidene difluoride membranes (Millipore). After blocking, the filters were incubated with the primary antibodies. Antibodies including, Akt, p-Akt, InsR (insulin receptor), p-InsR, Acc, p-Acc, HK2 (hexokinase 2), PFKL (6-phosphofructokinase, liver type), PKM1/2 (pyruvate kinase muscle isozyme), PDH (pyruvate dehydrogenase), PDHK1 (pyruvate dehydrogenase kinase isozyme 1) were purchased from Cell Signaling Technology. Antibodies, including PKLR (pyruvate kinase, liver and RBC), PEPCK (phosphoenolpyruvate carboxykinase), CPT1, PTBP1 were purchased from Abcam. Antibodies including GCK (glucokinase), PFK1 (phosphofructokinase 1), G6Pase (glucose-6-Phosphatase), PPARα, PPARδ, PPARγ were purchased from Santa cruz biotechnology. After washes and incubation with the appropriate horseradish peroxidase-conjugated secondary antibody (Santa Cruz Biotechnology), the immune complexes were visualized using a chemiluminescence reagent. Western blot results were densitometrically quantified with Quantity One software (Bio-Rad), and the intensity values were normalized to β-actin.

### Gene expression microarrays and data analysis

Total RNA was extracted from the frozen tissues by using TRIzol (Invitrogen). The RNA quantity and quality were measured with a NanoDrop ND-1000. RNA integrity was assessed by using standard denaturing agarose gel electrophoresis. The Rat 4 × 44 K Gene Expression Array was manufactured by Agilent. The updated content provided full coverage of rat genes and transcripts. Sample labeling and array hybridization were performed according to the Agilent One-Color Microarray-Based Gene Expression Analysis protocol (Agilent Technology). Briefly, the total RNA from each sample was linearly amplified and labeled with Cy3-UTP. The labeled cRNAs were purified with an RNeasy Mini Kit (Qiagen). The concentration and specific activity of the labeled cRNAs (pmol Cy3/μg cRNA) were measured with a NanoDrop ND-1000. Each labeled cRNA (1 μg) was fragmented in 11 μl of 10 × Blocking Agent and 2.2 μl of 25 × Fragmentation Buffer. Then, the mixture was heated at 60 °C for 30 min. Finally, 55 μl of 2 × GE Hybridization buffer was added to dilute the labeled cRNA. One hundred microliters of hybridization solution was dispensed into the gasket slide and assembled to create the gene expression microarray slide. The slides were incubated for 17 h at 65 °C in an Agilent Hybridization Oven. The hybridized arrays were washed, fixed and scanned with the Agilent DNA Microarray Scanner (part number G2505C). Agilent Feature Extraction software (version 11.0.1.1) was used to analyse the acquired array images. Quantile normalization and subsequent data processing were performed using the Gene Spring GX v12.1 software package (Agilent Technologies). After quantile normalization of the raw data, genes for which at least 46 out of 92 samples had Detected (“All Targets Value”) flags were chosen for further analysis. Differentially expressed genes with a statistically significant difference between the two groups were identified through Volcano Plot filtering. Differentially expressed genes between the two samples were identified through Fold Change filtering. GO and KEGG Pathway analyses were performed in the standard enrichment computation method.

### Real-time PCR

Total RNA from pancreas was isolated using TRIzol reagent (Invitrogen) according to the manufacturer’s protocol. First strand cDNA was synthesized using random primers and M-MuLV Reverse Transcriptase (New England Biolabs). PCR reactions were carried out with the manufacturer (Light Cycler 96, Roche), using the Quanti Tect SYBR Green RT-PCR Kit (QIAGEN). The following primers were used: Ins1, 5′-AGGACCCGCAAGTGCCACAA-3′ (sense) and 5′-TCCACAATGCCACGCTTCTGC-3′ (antisense); Ptbp1, 5′-AGTTCAAAGGTGATAACAGGAGCAC-3′ (sense) and 5′-CATGTAGGCCATGAGGTCCACCAC-3′ (antisense). The fluorescence curves were analyzed using LightCycler 96 software (Version 1.1).

### Clinical study

The study cohort consisted of 87 type 2 diabetic patients and included 38 women and 49 men who met the criteria based on the ‘Metabolic Surgery in the Treatment Algorithm for Type 2 Diabetes: A Joint Statement by International Diabetes Organizations’^6^. Patients were admitted and undergone routine physical examinations and systematic biochemical analyses before the surgery and were then followed up over two years. The examinations before and after surgery were performed. All patients undergone laparoscopic RYGB surgery at the Department of Surgery of the third affiliated hospital to the Third Military Medical University. All patients provided written informed consent. This study was approved by the ethics committee of the third affiliated hospital to the Third Military Medical University. The patients undergone oral glucose tolerance test (OGTT) to evaluate pancreatic β-cell function. OGTT (75-g glucose load, 2 h) were performed before surgery and follow-up over two years. All patients simultaneously undergone a complete evaluation of pancreas islet function using insulin and C-peptide release tests; plasma samples were collected at 0, 30, 60, and 120 min, and then assayed with a validated, highly sensitivity assay using a turbidimetric immunoassay (Orion Diagnostica, Espoo, Finland). The blood glucose concentrations were assayed using the glucose oxidase method^64^.

### Statistical analyses

All data were expressed as mean ± s.e.m. and *P* values were calculated using two-tailed Student’s *t*-test for pairwise comparisons, one-way ANOVA for multiple comparisons, and two-way ANOVA for multiple comparisons involving two independent variables. ANOVA analyses were subjected to Bonferroni’s post hoc test. Mann–Whitney non-parametric U test was used to analyse data in abnormal distribution. Correlation analysis was performed by Linear Regression Univariate Test. *P* < 0.05 was regarded as a significant effect or between-group difference. All tests were 2-tailed, and analyses were performed using either GraphPad Prism (GraphPad Software Inc., La Jolla, CA) or SPSS (version 12.0; SPSS, Inc., Chicago, IL).

## Electronic supplementary material

Supplementary Information

## Electronic supplementary material

**Supplementary Information** accompanies the paper at (10.1038/s41421-018-0062-x).
