# Supplementary material for: Non-insulin determinant pathways maintain glucose homeostasis upon metabolic surgery
Source: Cell Discov. 2018 Sep 25;4:58. doi: 10.1038/s41421-018-0062-x (PMC6155125; doi:10.1038/s41421-018-0062-x)
Supplement: Supplementary file 1 — Supplementary Information [file 41421_2018_62_MOESM1_ESM.pdf]

Supplementary Figure S1

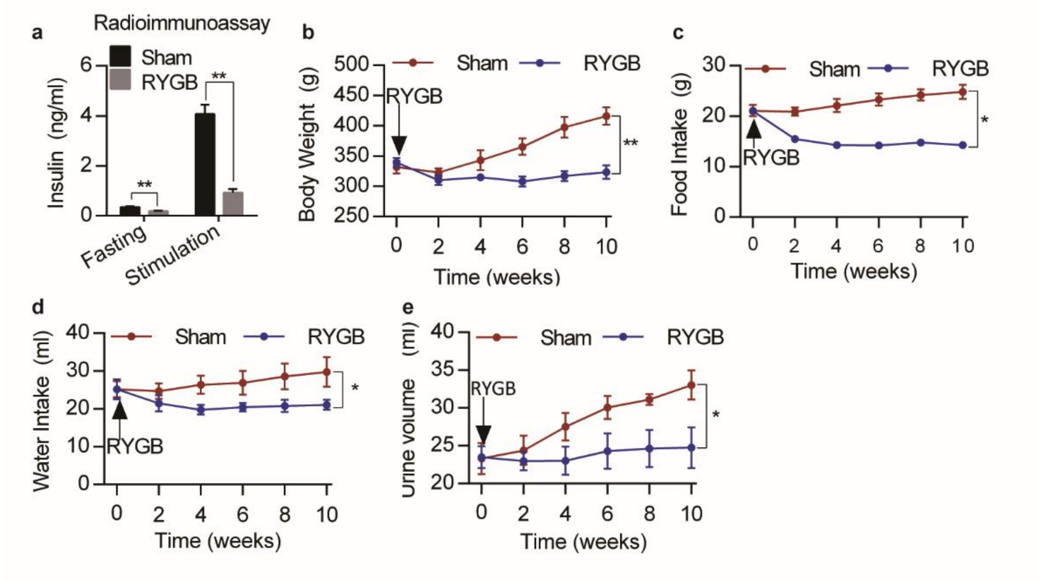

Supplementary Figure S1. Effect of RYGB on physiological parameters in rats. **(a)** Plasma insulin levels measured by radioimmunoassay in the fasting and glucose-stimulated states are significantly decreased in RYGB rats (n=10). **(b-e)** Body weight **(b)**, food intake **(c)**, water intake **(d)** and urine volume **(e)** of sham and RYGB rats (n=6-8). Values are shown as the mean ± s.e.m. Significance was calculated using two-way ANOVA with Tukey's post hoc analysis (a) and repeated-measures analysis (b-e). *P<0.05 and **P<0.01 compared with the sham group.

Supplementary Figure S2

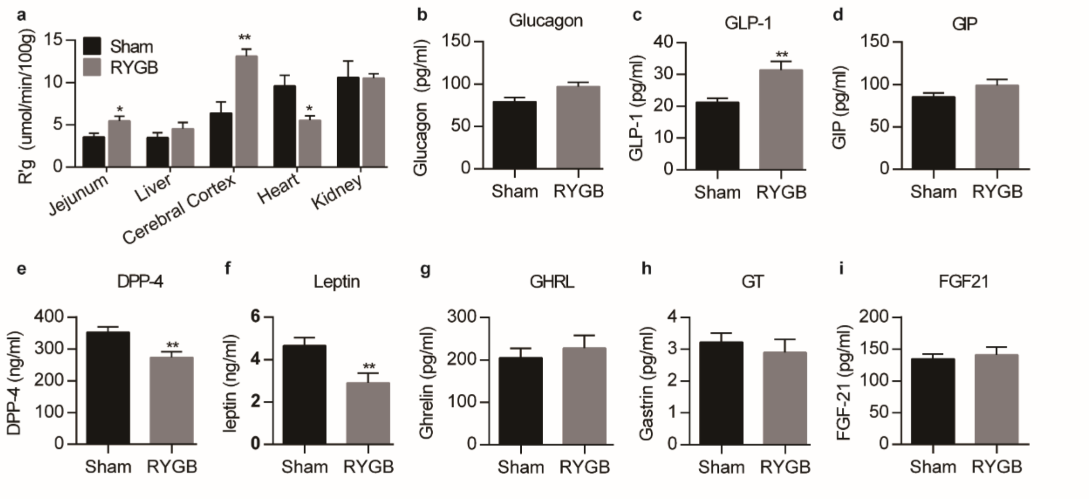

Supplementary Figure S2. Changes in gastrointestinal hormones and adipokines in plasma after RYGB. **(a)** Utilization of glucose in jejunum, liver, cerebral cortex, heart and kidney under hyperinsulinemic-euglycemic clamping homeostasis (n=4). **(b-i)** Plasma levels of gastrointestinal hormones and adipokines (glucagon, GLP-1, GIP, DPP-4, leptin, ghrelin, gastrin and FGF-21) in sham and RYGB rats (n=6-11). Values are shown as the mean ± s.e.m. Significance was calculated using two-way ANOVA with Tukey's post hoc analysis (a) and two-tailed unpaired t-test (b-i). **P*<0.05 and ***P*<0.01 compared with the sham group.

Supplementary Figure S3

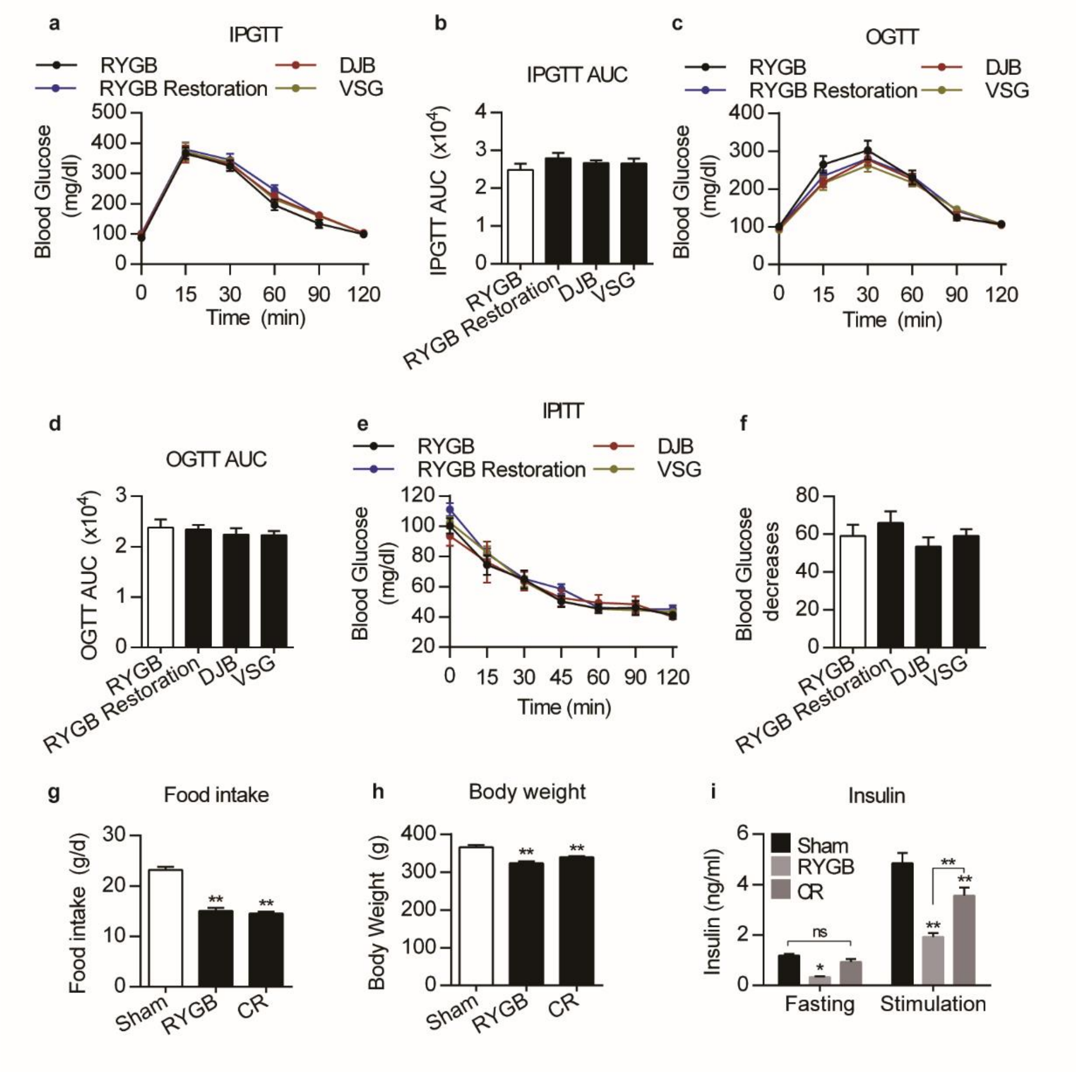

Supplementary Figure S3. Glucose and insulin tolerance tests in rats after different metabolic surgeries. **(a-f)** IPGTT (2g/kg) **(a-b)**, OGTT(2g/kg) **(c-d)** and IPITT **(e-f)** results in rats that underwent RYGB, DJB, VSG or RYGB restoration surgery. **(g-h)** Food intake, body weight and plasma insulin level in rats that treated with sham, RYGB or caloric restriction (CR). Values are shown as the mean ± s.e.m. Significance was calculated using two-way ANOVA with Tukey's post hoc analysis (b, d, f, i) and one-way ANOVA with Tukey's post hoc analysis (g, h). *P<0.05 and **P<0.01 compared with the sham group.

Supplementary Figure S4

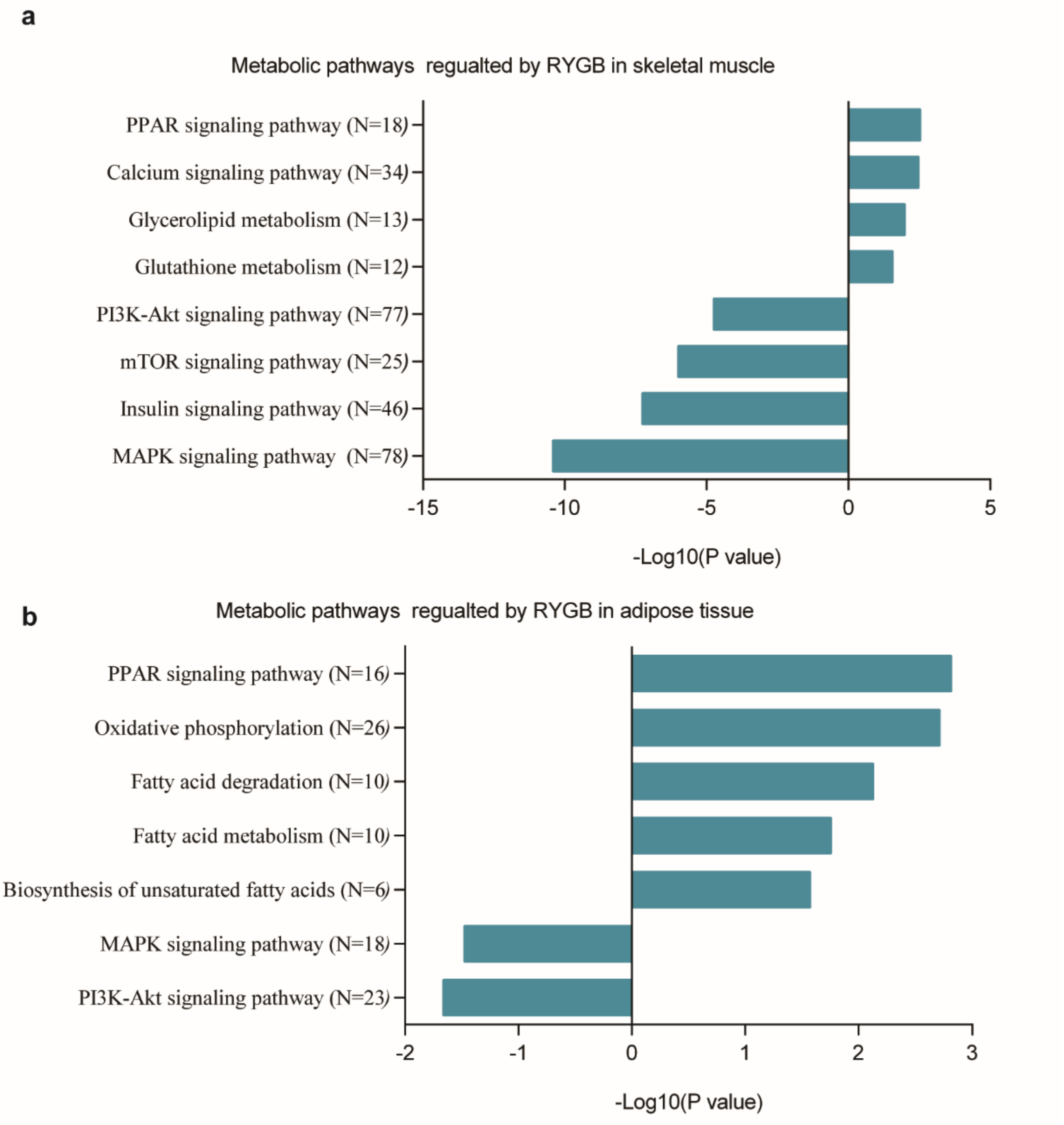

Supplementary Figure S4. Metabolic pathways in rat skeletal muscle and adipose tissue. **(a)** Enriched metabolic pathways in rat skeletal muscle regulated by RYGB (n=3). **(b)** Enriched metabolic pathways in rat adipose tissue regulated by RYGB (n=3).

Supplementary Figure S5

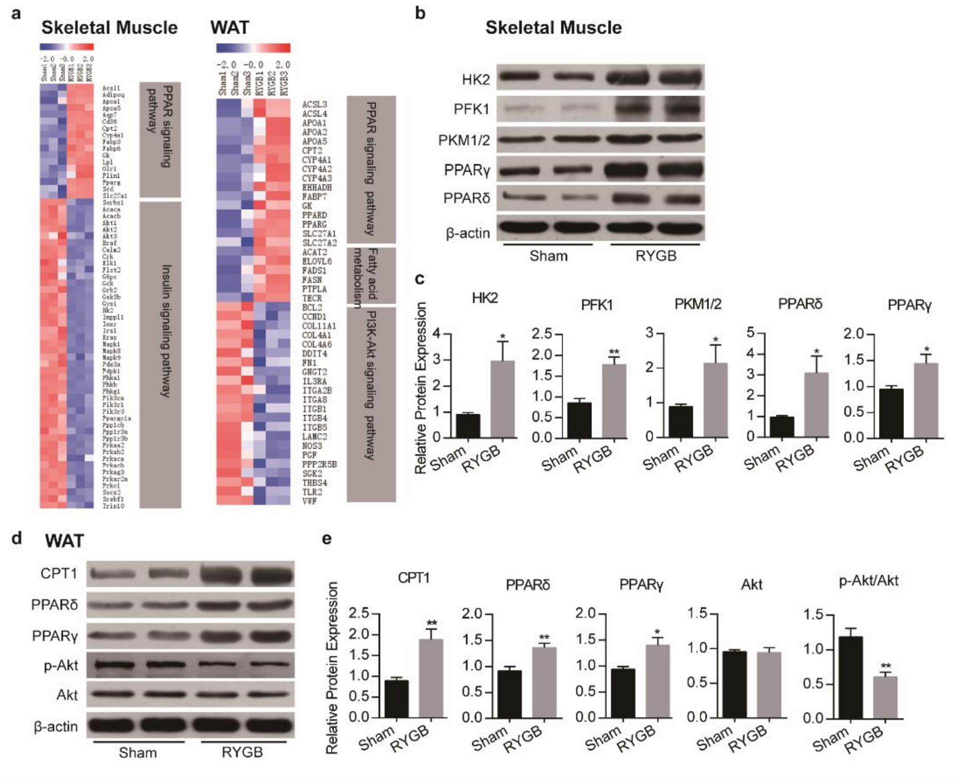

Supplementary Figure S5. Heatmap and related protein expression in skeletal muscle and WAT after surgery. **(a)** Expression levels of enriched metabolism pathway genes in skeletal muscle and WAT from sham and RYGB rats. **(b-c)** Immunoblots of HK2, PFK1, PKM1/2, PPARγ and PPARδ and β-actin in skeletal muscle from sham and RYGB rats (n=6). **(d-e)** Immunoblots of CPT1, PPARγ, PPARδ, p-Akt and Akt and β-actin in WAT from sham and RYGB rats (n=6). Values are shown as the mean ± s.e.m. Significance was calculated using a two-tailed unpaired t-test (c, e). **P*<0.05 and ***P*<0.01 compared with the sham group.

Supplementary Figure S6

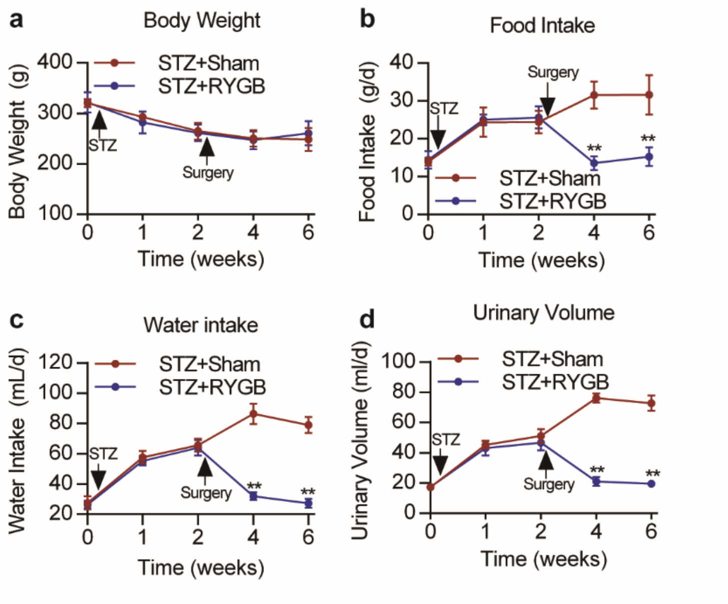

Supplementary Figure S6. Effect of RYGB on rats with islet disruption. **(a-d)** Body weight **(a)**, food intake **(b)**, water intake **(c)** and urine volume **(d)** of sham- or RYGB-treated STZ injected rats (n=6-8). Values are shown as the mean ± s.e.m. Significance was calculated using repeated-measures analysis (a-d). *P<0.05 and **P<0.01 compared with the sham group.

Supplementary Tables

Table S1

Baseline characteristics of patients with type 2 diabetes underwent metabolic surgery

	Pre-RYGB (n=87)	Post-RYGB	*p* value
Age (years)	42±11	—	—
Gender (male/female)	49/38	—	—
Duration(year)	5±4	—	—
BMI (kg/m^2)	30.8±2.3	23.4±1.2	0.00
WC (cm)	99.9±2.5	80.6±4.2	0.00
FBG (mmol/L)	8.1±0.6	6.2±0.8	0.00
HbA1c (%)	7.9±0.8	6.2±0.6	0.00
Fasting insulin (mIU/L)	20.1±2.5	12.4±1.6	0.01
SBP (mm Hg)	132±11	122±13	0.00
DBP (mm Hg)	80±11	72±10	0.00
Cholesterol (mmol/l)	4.99±0.25	4.12±0.21	0.00
Triglyceride (mmol/l)	3.06±0.24	1.34±0.17	0.00
HDL-C (mmol/l)	1.28±0.03	1.21±0.05	0.54
LDL-C (mmol/l)	2.70±0.06	2.42±0.07	0.89

The results are mean ± s.e.m. *p* values are obtained by paired two-tailed Student's t test. BMI, body mass index; FBG, fasting blood glucose; WC, waist circumference; SBP, systolic blood pressure; DBP, diastolic blood pressure.
